# Supplementary material for: PEA15 loss of function and defective cerebral development in the domestic cat
Source: PLoS Genet. 2020 Dec 8;16(12):e1008671. doi: 10.1371/journal.pgen.1008671 (PMC7723247; doi:10.1371/journal.pgen.1008671)
Supplement: S1 Table — Affected status is denoted by color with unaffected in white, obligate carriers based on breeding in grey, and affected cats in black. (PDF) [file pgen.1008671.s001.pdf]

S1 Table. Table of cats from the GM2A and MPSVI breeding colonies where the cerebral dysgenesis pathogenic variant was identified. Affected status is denoted by color with unaffected cats in white, obligate carriers based on breeding in grey, and affected cats in black.

Cat ID #	Sex	GM2A genotype	MPSVI genotype	Cerebral Dysgenesis Phenotype	PEA15 genotype	WGS ID	RNAseq ID	Amplicon ID
2913	male	unknown	1	Unaffected	0 or 1 (Imputed)			
R22	male	1	0	Unaffected	0 (Imputed)			
R21	female	1	0	Unaffected	0 (Imputed)			
596	female	0	0	Unaffected	0 or 1 (Imputed)			
B15 (564)	female	0	unknown	Unaffected	1 (Imputed)			
R24	male	1	0	Unaffected	0 (Imputed)			
B17	female	0	1	Unaffected	1 (Imputed)			
B39	male	unknown	unknown	Unaffected	1 (Imputed)			
R23	male	1	0	Unaffected	0 (Imputed)			
BXR 261	female	0	1	Unaffected (obligate carrier)	1			SAMN10276058
B88	female	unknown	unknown	Unaffected	1 (Imputed)			
10-263	male	0	1	Unaffected (obligate carrier)	1	SAMN10276158		SAMN10276070
10-267	female	1	0	Unaffected (obligate carrier)	1		SAMN10276168	SAMN10276084
851	male	1	1	Unknown (early death)	2			SAMN10276104
854	male	0	1	Unaffected	1			SAMN10276116
10-717	female	0	unknown	Unaffected (obligate carrier)	1 (imputed)			
752	female	0	0	Unaffected	1			SAMN10276097
736	male	0	0	Unaffected	1			SAMN10276061
751	female	0	2	Unaffected	1			SAMN10276085
753	male	0	2	Unaffected	1			SAMN10276109
772	female	0	0	Unaffected	1			SAMN10276066
773	female	0	1	Unaffected	0			SAMN10276122
10-670	female	0	1	Unaffected (obligate carrier)	1	SAMN10276154	SAMN10276172	
492	female	unknown	1	Unaffected	0 (Imputed)			
10-820	male	0	1	Unaffected	1			SAMN10276095
10-737	female	0	2	Affected	2			SAMN10276119
10-498	male	0	0	Unaffected	1			SAMN10276130
10-499	female	0	1	Affected	2		SAMN10276171	SAMN10276142
858	male	0	0	Unaffected	1			SAMN10276140
860	male	0	2	Unaffected	1			SAMN10276152
10-494	male	0	0	Unaffected	0			SAMN10276082
10-496	male	0	1	Unaffected	1			SAMN10276106
10-738	male	0	1	Unaffected	0			SAMN10276131
10-739	female	0	1	Unknown (early death)	1			SAMN10276143
857	male	0	0	Unaffected	0			SAMN10276135
10-818	male	0	1	Unknown (early death)	0			SAMN10276071
10-819	male	0	1	Unknown (early death)	2			SAMN10276083
10-495	female	0	1	Unaffected	1			SAMN10276094
10-740	male	0	1	Unaffected	1			SAMN10276060
863	female	0	0	Unknown (early death)	2			SAMN10276064
10-821	male	0	1	Affected	2			SAMN10276107

10-497	female	0	1	Unaffected	1			SAMN10276118
10-741	male	0	2	Unaffected	1			SAMN10276072
10-471	male	1	0	Unaffected (obligate carrier)	1		SAMN10276169	SAMN10276096
10-822	male	0	2	Affected	2			SAMN10276150
10-487	male	0	2	Affected	2			SAMN10276108
823	male	0	0	Unaffected	1			SAMN10276067
10-759	male	0	2	Unaffected	1			SAMN10276062
10-760	male	0	2	Unaffected	1			SAMN10276074
10-758	male	0	0	Affected	2			SAMN10276137
825	male	0	1	Unaffected	1			SAMN10276079
10-488	female	0	0	Affected	2			SAMN10276101
10-757	female	0	2	Unaffected	1			SAMN10276145
10-490	male	0	1	Unaffected	1			SAMN10276120
10-732	female	0	1	Unaffected (obligate carrier)	1	SAMN10276157	SAMN10276174	
10-733	male	0	0	Affected	2			SAMN10276144
10-480	female	0	1	Unaffected	1			SAMN10276089
10-481	male	0	1	Affected	2	SAMN10276156	SAMN10276173	
761	female	0	1	Unaffected	1			SAMN10276149
10-730	female	0	0	Affected	2	SAMN10276155		
763	female	0	1	Unaffected	0			SAMN10276098
10-479	female	0	1	Unaffected	1			SAMN10276077
762	male	0	1	Unaffected	1			SAMN10276086
764	female	0	1	Unaffected	1			SAMN10276110
493	female	0	1	Unaffected (obligate carrier)	1		SAMN10276170	SAMN10276132
840	male	0	1	Unaffected	0			SAMN10276087
841	male	0	1	Unaffected	1			SAMN10276099
10-817	female	0	0	Unaffected	0			SAMN10276059
10-746	female	0	0	Unaffected	0			SAMN10276125
10-780	male	1	0	Unaffected	1			SAMN10276102
10-781	male	1	0	Unaffected	0			SAMN10276134
10-782	female	1	0	Unaffected	1			SAMN10276146
10-783	female	2	0	Affected	2			SAMN10276114
10-742	female	1	0	Affected	2		SAMN10276162	
10-743	male	2	0	Affected	2			SAMN10276113
10-745	male	2	0	Affected	2			SAMN10276073
10-779	male	0	0	Affected	2			SAMN10276090
10-774	male	0	0	Affected	2		SAMN10276163	
10-776	female	0	0	Unaffected	0			SAMN10276078
804	female	0	0	Unaffected	1			SAMN10276075
805	female	0	1	Unaffected	0			SAMN10276138
754	male	0	0	Unaffected	1			SAMN10276121
756	male	0	0	Unaffected	1			SAMN10276133
10-791	female	0	1	Unknown (early death)	2			SAMN10276126
10-793	male	0	2	Unknown (early death)	2			SAMN10276063
855	male	0	1	Unaffected	1			SAMN10276128
859	male	0	1	Unaffected	1			SAMN10276147
10-792	male	0	0	Affected	2		SAMN10276161	
844	male	0	0	Unaffected	0			SAMN10276151
847	female	0	0	Unaffected	1			SAMN10276111

845	male	0	1	Unaffected	0			SAMN10276068
10-848	female	0	0	Unaffected (obligate carrier)	1			SAMN10276080
10-850	female	0	0	Unaffected	1			SAMN10276123
10-849	male	0	0	Unaffected (obligate carrier)	1			SAMN10276092
10-829	male	0	0	Affected	2			SAMN10276091
10-830	male	0	0	Affected	2			SAMN10276103
10-831	female	0	unknown	Unaffected	1			SAMN10276115
10-837	male	0	1	Unaffected	1			SAMN10276153
10-839	female	0	1	Unaffected	1			SAMN10276139
10-838	male	0	1	Unaffected (obligate carrier)	1			SAMN10276141
10-836	male	0	1	Unaffected (obligate carrier)	1			SAMN10276127
10-874	male	0	0	Unaffected	1 (imputed)			
877	male	0	0	Affected	2		SAMN10276160	
878	male	0	0	Unaffected	1 (imputed)			
879	male	0	0	Affected	2		SAMN10276159	
880	male	0	1	Unknown (early death)	2			SAMN10276069
881	female	0	0	Unaffected	0			SAMN10276081
882	female	0	1	Affected	2			SAMN10276093
10-883	female	0	1	Unaffected	0			SAMN10276105
10-885	female	0	0	Unaffected	1			SAMN10276076
912	female	0	0	Unaffected	0			SAMN10276136
903	female	0	0	Unaffected	1			SAMN10276148
904	female	0	0	Unaffected	1			SAMN10276112
909	female	0	0	Unaffected	0			SAMN10276124
908	male	0	0	Affected	2			SAMN10276065
894	female	0	1	Unaffected	1			SAMN10276117
897	male	0	1	Unaffected	1			SAMN10276129
891	female	0	1	Affected	2			SAMN10276088
10-892	female	0	1	Affected	2			SAMN10276100
11-1044	female	0	0	Unaffected	0		SAMN10276164	
8-1344	male	0	0	Unaffected	0		SAMN10276165	
7-747	male	0	0	Unaffected	0		SAMN10276166	
7-734	female	0	0	Unaffected	0		SAMN10276167	
