# Supplementary material for: PEA15 loss of function and defective cerebral development in the domestic cat
Source: PLoS Genet. 2020 Dec 8;16(12):e1008671. doi: 10.1371/journal.pgen.1008671 (PMC7723247; doi:10.1371/journal.pgen.1008671)
Supplement: S2 Table — Cerebrospinal fluid protein concentration and cell counts in affected and carrier cats with a reference interval based on normal cats that was established by the Auburn University small animal teaching hospital. (PDF) [file pgen.1008671.s002.pdf]

S2 Table. Cerebrospinal Fluid Analysis. Cerebrospinal fluid protein concentration and cell counts in affected and carrier cats with a reference interval based on normal cats that was established by the Auburn University small animal teaching hospital.

Analyte	Affected	Carrier	Reference Interval
Protein (mg/dL)	12 ± 2.65	12.5 ± 3.54	< 30
Nucleated cells (cells/uL)	0	0	0 - 8
Erythrocytes (cells/uL)	0	0	0
