# Supplementary material for: PEA15 loss of function and defective cerebral development in the domestic cat
Source: PLoS Genet. 2020 Dec 8;16(12):e1008671. doi: 10.1371/journal.pgen.1008671 (PMC7723247; doi:10.1371/journal.pgen.1008671)
Supplement: S3 Table — Merlin 1.1.2 was used for haplotyping and LOD score calculation. LOD score was calculated using parametric linkage analysis assuming a rare recessive model. Each marker is considered independently, equivalent to a theta value of zero. Note that, while we focused genotyping on chromosome F1 based on homozygosity mapping, we selectively genotyped a handful of markers on other chromosomes. (PDF) [file pgen.1008671.s003.pdf]

S3 Table. Haplotyping and LOD Score Calculation. Merlin 1.1.2 was used for haplotyping and LOD score calculation. LOD score was calculated using parametric linkage analysis assuming a rare recessive model. Each marker is considered independently, equivalent to a theta value of zero. Note that, while we focused genotyping on chromosome F1 based on homozygosity mapping, we selectively genotyped a handful of markers on other chromosomes.

Marker (FelCat8 Chr, Pos)	LOD Score	On Figure	PEA15 Variant
chrA1 121761499	-0.243		
chrA1 136687002	1.778		
chrA2 502614	-5.345		
chrB1 201861287	1.098		
chrB3 39796402	-3.691		
chrC1 23743729	-3.304		
chrC1 23802591	-3.315		
chrC2 2270141	-3.886		
chrC2 8832783	-1.492		
chrC2 11824978	0.491		
chrD1 101302305	-0.422		
chrD1 105492266	-1.688		
chrD3 16631287	-0.189		
chrD4 1447611	-3.588		
chrD4 1888073	-2.335		
chrD4 2172964	-0.917		
chrD4 57669950	-3.952		
chrE1 5662690	-8.844		
chrE2 6093703	-2.782		
chrE3 28445872	-2.298		
chrF1 1958897	-0.207		
chrF1 30832600	-5.767	Yes	
chrF1 45094240	-3.188	Yes	
chrF1 54911523	-1.667	Yes	
chrF1 60004768	0.931	Yes	
chrF1 60937957	1.689	Yes	
chrF1 62825737	2.996	Yes	
chrF1 63114138	3.092	Yes	
chrF1 63491285	3.871	Yes	
chrF1 64027614	4.646	Yes	
chrF1 64508880	6.225	Yes	
chrF1 64651564	8.288	Yes	
chrF1 64739960	8.314	Yes	
chrF1 64746818	8.316	Yes	
chrF1 64831767	9.905	Yes	
chrF1 64862420	10.041	Yes	
chrF1 64927620	10.005	Yes	
**chrF1 65219219**	**10.104**	**Yes**	Yes
chrF1 65287523	10.104	Yes	
chrF1 65319044	10.104	Yes	
chrF1 65547493	5.986	Yes	
chrF1 65559266	5.969	Yes	
chrF1 66054190	5.095	Yes	
chrF1 66460118	1.469	Yes	
chrF1 66897299	-2.87	Yes	
chrF1 67340525	-1.91	Yes	
chrF1 67595441	-2.763	Yes	
chrF1 67821165	-2.683	Yes	
chrF1 69294593	-2.287	Yes	
chrF1 69907283	-1.348	Yes	
