# Supplementary material for: PEA15 loss of function and defective cerebral development in the domestic cat
Source: PLoS Genet. 2020 Dec 8;16(12):e1008671. doi: 10.1371/journal.pgen.1008671 (PMC7723247; doi:10.1371/journal.pgen.1008671)
Supplement: S4 Table — These 10 variants met initial zygosity filtering criteria from WGS and RNA-seq data. (PDF) [file pgen.1008671.s004.pdf]

S4 Table. Variants from Figure 3A. These 10 variants met initial zygosity filtering criteria from WGS and RNA-seq data.

Chromosome	FelCat9 Position	Reference	Alternate
chrF1	65397512	C	T
chrF1	65397980	C	T
chrF1	65398081	C	G
chrF1	65398552	A	C
chrF1	65572059	G	A
chrF1	65579963	C	T
chrF1	66766618	A	G
chrF1	66768209	G	A
chrF1	66768323	GT	G
chrF1	69703441	A	G
