# Supplementary material for: PEA15 loss of function and defective cerebral development in the domestic cat
Source: PLoS Genet. 2020 Dec 8;16(12):e1008671. doi: 10.1371/journal.pgen.1008671 (PMC7723247; doi:10.1371/journal.pgen.1008671)
Supplement: S6 Table — Note that multiple primer pairs were included for the top nominated region to maximize genotyping success and increase coverage. The PEA15 frameshift site is bolded. (PDF) [file pgen.1008671.s006.pdf]

S6 Table. Primer pairs for amplicon sequencing. Note that multiple primer pairs were included for the top nominated region to maximize genotyping success and increase coverage. The *PEA15* frameshift site is bolded.

FelCat8 Chr, Pos	Pair	Forward Primer	Reverse Primer
chrA1 121761499	1	TGGACCTACAGAAGGGAGAAGG	AAAGTGTTAGGCCATTTCTTGGCCTA
chrA1 136687002	1	AGGTAAAAGTACTCCTGATGCTGAAG	TAAAAAATACAAACATTAGTGTTTATATGGTCAGG
chrA2 502614	1	CTCACTATGCGGGATATAAGTACCTA	TGGGGTCTATAGGCACAATGTAATTTC
chrB1 201861287	1	GACCTGTGTGCACATGGGG	GGCTCAGAACGCAGCTACG
chrB3 39796402	1	AGAACAGTGACATTAACTCACAAGAGATTCT	TTGGCACACATCCCAGGGCC
chrC1 23743729	1	CCTGCAGCTCGCCCGTTC	TTCTCCCTCATCCCGCCGATC
chrC1 23802591	1	AACTGCCCACCTGCCTCCTTC	AGAAGTGGAAGAAAGAGTAGCTGTAGGT
chrC2 2270141	1	GCTCCTAACGTAAGCCTTATTTTGTTTAAC	TTTTTATGGTTCGTTTTCTCCAACTGATTTAACAA
chrC2 8832783	1	ACGCATGTGCCCCCAGGTG	GCTCCCGTTCTCGCAGCCAAA
chrC2 11824978	1	CAGAGCCTTAGGCCGAGGC	GTGCTCTGCCTGGCGTGG
chrD1 101302305	1	CCCAAGGAGTAGCTGGAACTAG	AGGCTCTCATGCTCTGACACAC
chrD1 105492266	1	GAAGTCCTTGGCTGGCAGCT	CTCAGACACAGGGAGACTTCC
chrD3 16631287	1	ATCAGTGTCCTCAGAGCAGAGG	TATCTCTCCTCTCCCTTCTGTCTTTT
chrD4 1447611	1	CTTGTGGGCCACTGTCCTCA	GCCCCTACTTACAGGTAGACTTC
chrD4 1888073	1	GGCTTCAAGTCGCTTCTCACCA	AAGCCTCGGCCACACTCCC
chrD4 2172964	1	CGGCTCCATCTCGCCCGCA	GTTGAGCACTGGGTGGGTGTAGT
chrD4 57669950	1	AATGTGATCAGCATGGTTGTGGCTAAT	TTATCTGAAAGGTCCTGCTTATCTTCC
chrE1 5662690	1	GGTCATCACAGTGTGACTTGCGT	TAAACGAGAAATGCGACGCCAGC
chrE2 6093703	1	ATCCCAGCTCGATCTTCATAAGTGT	ATTGGTAACTGTATGTTGTAGAAGTACATACA
chrE3 28445872	1	CAGGTGGACTACAATGCCAGTG	CCCACACTGCCCTCTCCC
chrF1 1958897	1	GTGTTCGGATACAGGAGGCCTC	GGGTACTGGGCATGGGGAAG
chrF1 30832600	1	ACAGCAATACTTCTTCAGTTTTTAAAGTGTC	TGTAGCGGAGAAACACAGCGC
chrF1 45094240	1	ACTATCTTTGTGGTGTGCCAATCTAAAAT	TATGAACTCCTGTTTCAGATGTTTGGAAA
chrF1 54911523	1	AGCCAACCAACCACCAACCATC	GAAACTGTATGATGACTGCTCCAGC
chrF1 60004768	1	TAGTATGAATGCAAGTGAACTTAATTATGCATTC	AAATAACCTAGAGAATCTTTTTGTTTTGCTTATTC
chrF1 60937957	1	GCCACTCCAGCTCCACGTCTAT	ACGGGCACCTACCTGAGAGCT
chrF1 62825737	1	GATAAACTTCTAAAACATGTATGGATAATATGAAG	ACCTACCTCTTTAGGAGCCTCC
chrF1 63114138	1	AGCCAGGAATTTGTGAGCTGAACAAA	TAAAAGTCATACAATTGTCTTCTCCTGTTTCA
chrF1 63491285	1	AGAGTGGAGCCTGAGGGGG	GTCACTGCCCTGTGCTCTCTTC
chrF1 64027614	1	CACGCAGCCTCAGATTCTACTC	TTGTTTGGATTTTCCCCAGCAAATGGAA
chrF1 64508880	1	CTGCCCGAAGGGGTCCTCTG	GCCGGACCCGAGAGCCAC
chrF1 64651564	1	TCTTGGTGTCAGGGGTCGTGAG	GCTCAAGCCCCTGCTAGAGC
chrF1 64739960	1	TGGGCCGTAGGTGCAGGTG	GCTCTCCCAAGGGTATGAGAAG
chrF1 64746818	1	AGCCTATCTTCTTGCTTCCCTATTC	GCTTCTGAATCCCATGGGGC
chrF1 64831767	1	CCTAGCCCCCTTTGTATTTTGTTAG	AAGCACATTACTATGGATCTCCTTTGG
chrF1 64862420	1	TGTCAGCTCTGTCTGACACTGAC	TCTCTCTCTCTCTCCTTGGGCT
chrF1 64927620	1	ACGGAGGTACCTCCCTGTGC	CTGGGTCTGGGGCATCTGC
**chrF1 65219219**	1	GGGTCAGCTTAGTGTCCAGCT	TCAGCAGCCCCGCCCCTT
chrF1 65287523	1	GAATTGAAGGGGATCTCCGCCAC	TGGGGCGCCTCCGCACCT
chrF1 65319044	1	ACTGGGGTGTCACTCGGACAGCAG	CCGCCCACCAGCCGGGCA
chrF1 65547493	1	AGGATGCCTTTCTAGTGTCAGGGG	TGGAGATCGCTCTGGCCTTCG
chrF1 65559266	1	GGCCCCAAACTTCCCCTTTACATT	CACTCCCAGCTACCGCCATGA
chrF1 66054190	1	CCGCCACTGGAAGAGAGGC	GAGGACGCTACCAAGTTCACCTT
chrF1 66460118	1	ACAGAGACTGCACTGATATTCGGA	TTCGGCTGAAGGGATCTTGAAGA
chrF1 66897299	1	GCACACAGAGACTTGCAAATCCATC	TCTTGCGTTCTGGCTCTGGAAG
chrF1 67340525	1	GTCACAAGGTCACTAAAGACCGAG	GGGCGTTGTCCTCGCGATAAAA
chrF1 67595441	1	ACCCTCGCCCATCCCCAGA	TACTGGTCAGCCCCGGGTC

chrF1 67821165	1	GAAACAGCCATAGAGGTCAAAATGGT	TAAGTAGCTCTAGAGTCTCTTTTTCTTGA
chrF1 69294593	1	ACCTCCGCCCCAACTACTGG	CTCTTCCTGTGGATCTTTGTCTTCGT
chrF1 69907283	1	TTCCTGGAGGAAGCAAGGCAG	CGCATCTAGCGGACAGGGC
chrF1 64027614	2	TACCTTTAGCGGGTGCAGGATG	GAATGACTCCAAAGAAAAAAATTCAGATGACTT
chrF1 64508880	2	GTCCTCTGGGGCGAGGGC	AGCCACCTGTTAATGTCCAGCCC
chrF1 64651564	2	GGTCGTGAGTTCGATCCCCG	CTAGAGCCTTGCTCACCGGCTT
chrF1 64739960	2	GCAGGTGCATTCCTCAGCATGA	GTATGAGAAGCTGGACACTCTTCTC
chrF1 64746818	2	TACCTGTGTCCTGCCAGAACCC	CCATCACCATCTACTGGATGCC
chrF1 64831767	2	GTATTTTGTTAGCCAAACGCTTGTAGC	TGGATCTCCTTTGGAAATGGTCTC
chrF1 64862420	2	TGACACTGACCTCGCTCTGCC	CCTTGGGCTGGCTGAGTCCT
chrF1 64927620	2	CCTGTGCCCTCTACAGAACCAT	ATCTGCTGGAACACATGATGTGGG
**chrF1 65219219**	2	GTCCAGCTCATCCTCCTCGGAG	CCCTTCCAGGGGCGTGCC
chrF1 65287523	2	TCTCCGCCACCTTGGGGTTTC	CACCTGCCCACCTTGCTGAC
chrF1 65319044	2	TCGGACAGCAGTCCCGACC	GGGCAGGTGCAGGGCTCA
chrF1 65547493	2	AGTGTCAGGGGTACTCACCGTG	GGCCTTCGGAGGAGCTCGT
chrF1 65559266	2	CCCTTTACATTGGCAAAAATGTCCAGTTT	CGCCATGACAGAACCCCCGA
chrF1 66054190	2	GAGAGGCCTGAGAAGCGCG	AAGTTCACCTTGGGAGAAGATATTTGG
chrF1 66460118	2	CTGATATTCGGAGGCTCCTTCTG	GATCTTGAAGACAGTGGACAGAATGAAAA
chrF1 66897299	2	TGCAAATCCATCACCACTGAGCTG	CTCTGGAAGCGCTGGTAAGTCT
chrF1 67340525	2	TAAAGACCGAGAAAAACGGTTTCTGGTTT	GCGATAAAACCGGTACAGGATGG
chrF1 67595441	2	CCCAGAGGATCTGCGAGGTGT	CGGGTCCTGCACAGCTCCA
chrF1 64831767	3	CCAAACGCTTGTAGCACCAGTG	GGAAATGGTCTCGAGTCCCCAT
chrF1 64862420	3	CGCTCTGCCGTGGCATCTTC	TGAGTCCTGGCAGGTAATGGTGG
chrF1 64927620	3	ACAGAACCATCCTGGGTGCCG	ACATGATGTGGGAAGCTCAGTGTC
**chrF1 65219219**	3	CTCCTCGGAGATCTTCAGAACAC	CGTGCCCTCTGATAGCCAGTGT
chrF1 65287523	3	TGGGGTTTCTATCCCTCATCTTCC	TTGCTGACGCCCAGCTCCC
chrF1 65319044	3	CCCGACCCCACACCCCTCT	GGCTCAGCTCCCCTTCCTCG
chrF1 65547493	3	ACTCACCGTGCACCTTGAGCT	AGCTCGTGGCCACGTTTTTCC
chrF1 65559266	3	GCAAAAATGTCCAGTTTCATCCAAGAGTA	ACCCCCGACTGGCTTCTCTC
