# Supplementary material for: PEA15 loss of function and defective cerebral development in the domestic cat
Source: PLoS Genet. 2020 Dec 8;16(12):e1008671. doi: 10.1371/journal.pgen.1008671 (PMC7723247; doi:10.1371/journal.pgen.1008671)
Supplement: S1 Fig — Phenotype is denoted by color and indicated on the pedigree with unaffected in white, obligate carriers with a central dot, and homozygous mutant cats in black. (PDF) [file pgen.1008671.s007.pdf]

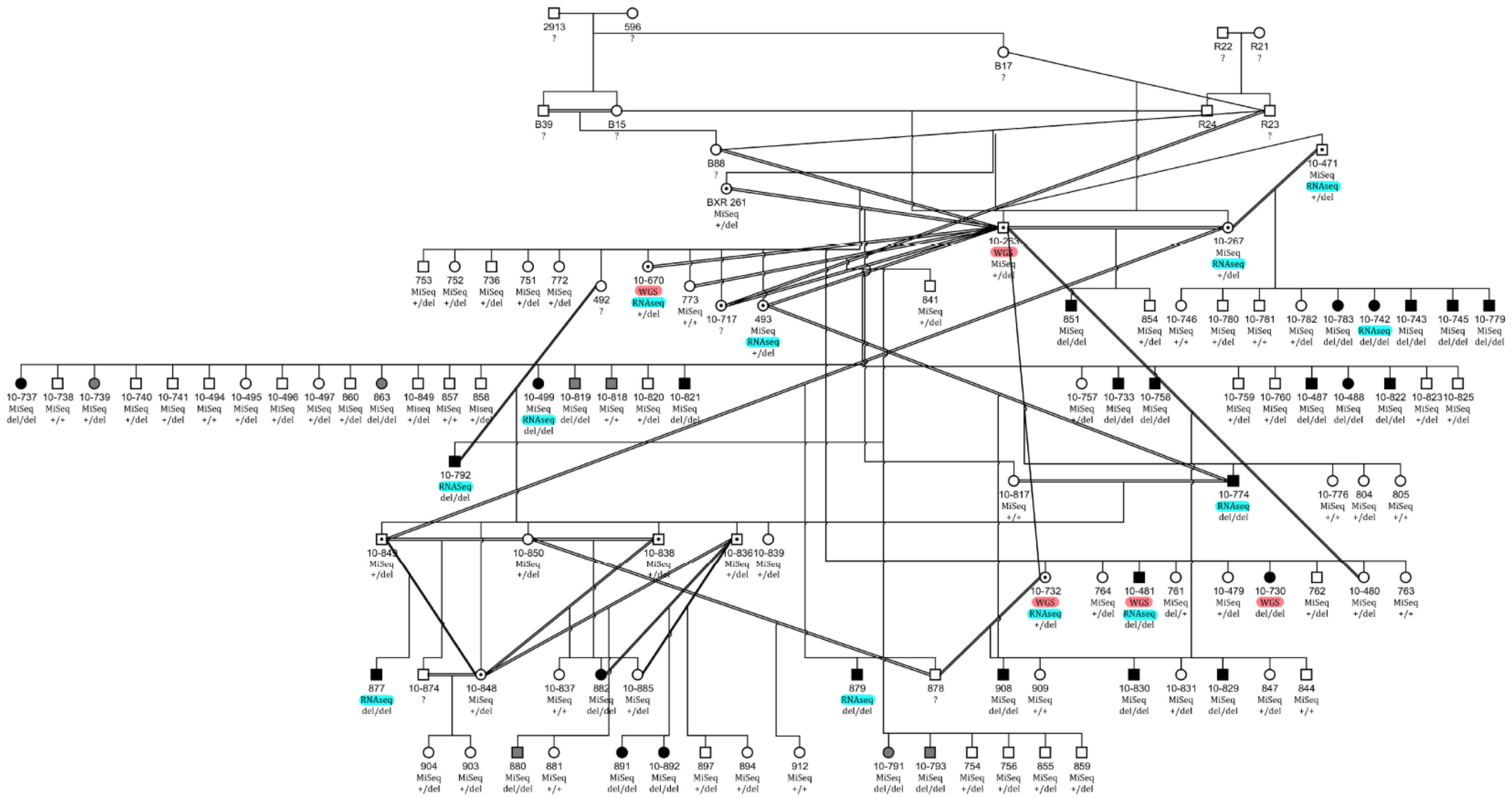

S1 Fig. Pedigree. Circles indicate females, squares indicate males. Phenotype is denoted by colored/marked symbols: unaffected animals have white filled symbols, obligate carriers have a white filled symbol with a central dot, and homozygous affected cats have black filled symbols. Grey filled symbols indicate an early death with an unknown phenotype. Sequencing approach and genotype, when known, is indicated below each symbol. RNAseq samples are highlighted in blue, whole genome sequencing samples are highlighted in red. A "?" below a symbol indicates an unknown genotype. All of the cats in this pedigree are deceased, except for 10-892.
