# Supplementary material for: PEA15 loss of function and defective cerebral development in the domestic cat
Source: PLoS Genet. 2020 Dec 8;16(12):e1008671. doi: 10.1371/journal.pgen.1008671 (PMC7723247; doi:10.1371/journal.pgen.1008671)
Supplement: S2 Fig — (A) Changes in aspartate amino transferase and (B) lactate dehydrogenase enzyme activity in adult cats (n = 3) from the cerebral dysgenesis cohort. (PDF) [file pgen.1008671.s008.pdf]

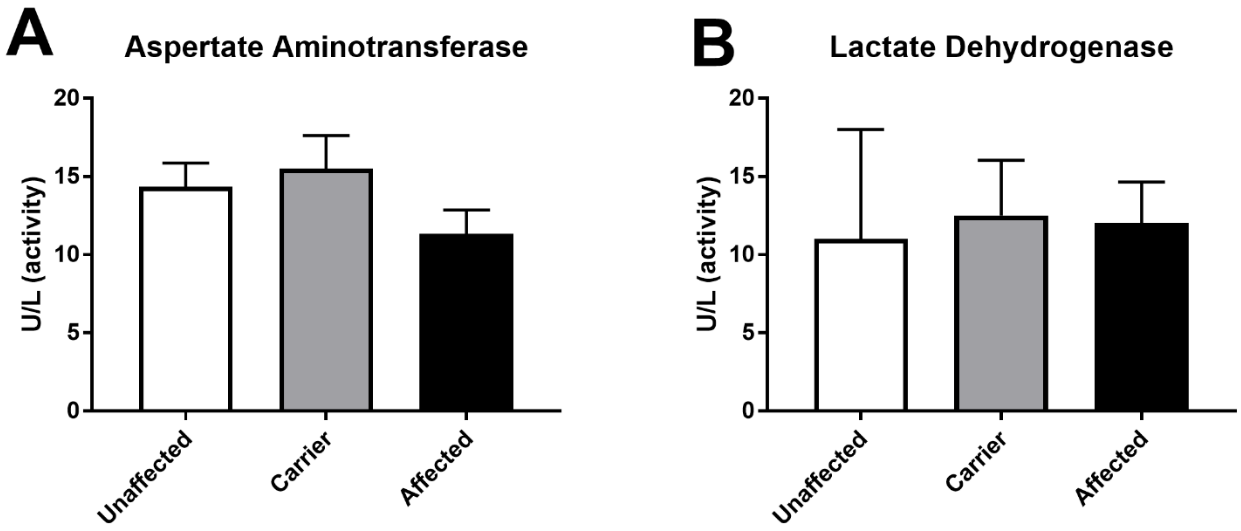


**S2 Fig. Cerebrospinal fluid enzyme activity.** (**A**) Changes in aspartate amino transferase and (**B**) lactate dehydrogenase enzyme activity in cerebrospinal fluid from adult cats (n = 3 per group).
