# Supplementary material for: PEA15 loss of function and defective cerebral development in the domestic cat
Source: PLoS Genet. 2020 Dec 8;16(12):e1008671. doi: 10.1371/journal.pgen.1008671 (PMC7723247; doi:10.1371/journal.pgen.1008671)
Supplement: S3 Fig — (A) When averaged over 10,000 base pair windows, coverage stays above 30x for all cats across the window linked to the phenotype. (B) When averaged over 1,000 base pair windows, only a few regions dip below 20x coverage. (C) Regions with less than 20x coverage in all 5 cats. Note that regions are either in repetitive intronic or intergenic regions. (PDF) [file pgen.1008671.s009.pdf]

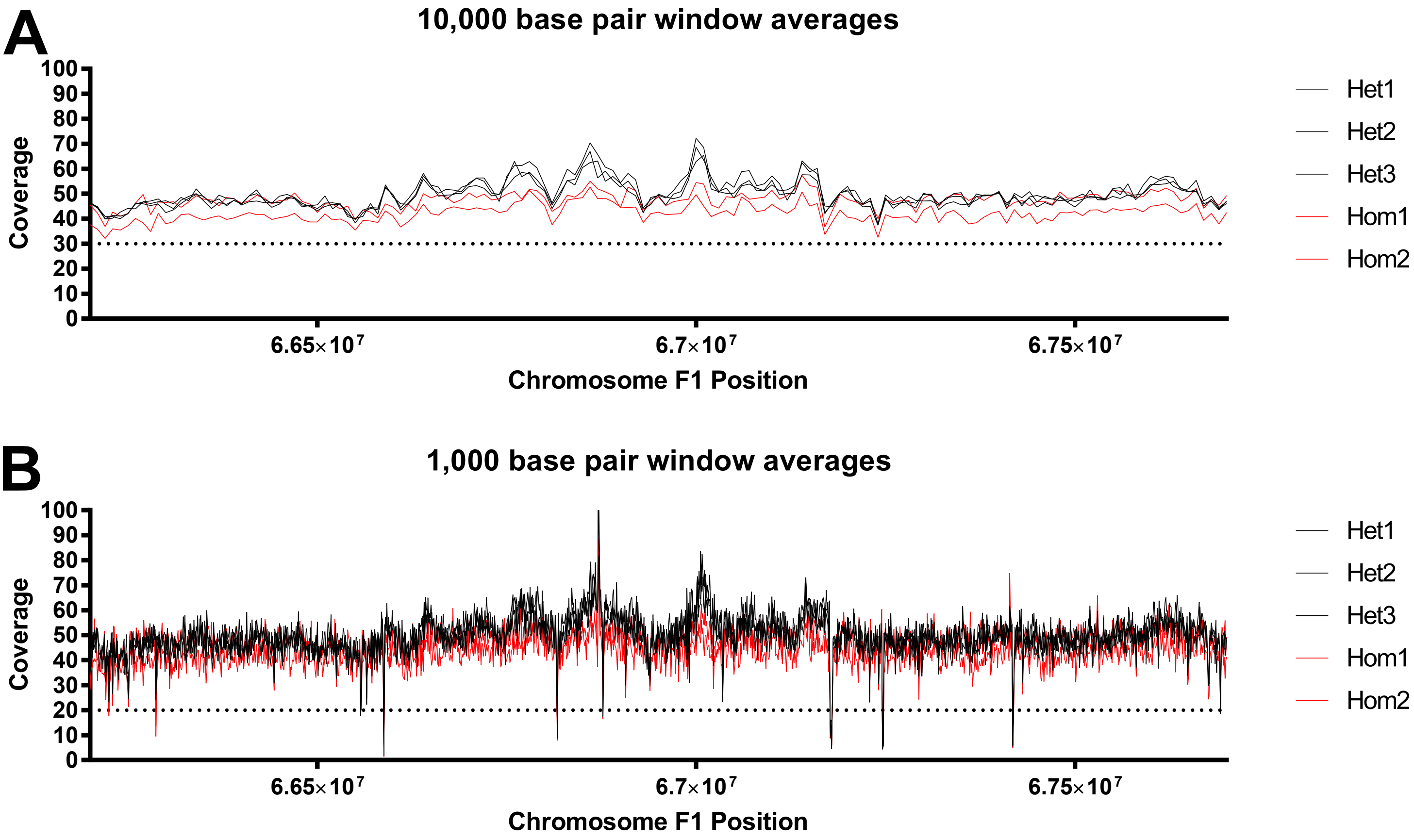

C

Position	Coverage	Location	Characteristics	Brain Expression
66588000	3.006	Intron of Vangl2	repetitive/ga-rich	Low
66817000	10.61	intron of Atp1a4	repetetive/gc-rich	None
67177000	15.3	Intergenic	repetitive	N/A
67178000	16.12	Intergenic	repetitive	N/A
67179000	8.914	Intergenic	repetitive	N/A
67246000	6.045	Intergenic	repetitive	N/A
67247000	7.547	Intergenic	repetitive	N/A
67418000	9.191	Intergenic	repetitive	N/A
