# Supplementary material for: PEA15 loss of function and defective cerebral development in the domestic cat
Source: PLoS Genet. 2020 Dec 8;16(12):e1008671. doi: 10.1371/journal.pgen.1008671 (PMC7723247; doi:10.1371/journal.pgen.1008671)
Supplement: S4 Fig — (A) Conservation of PEA15 gene sequence was performed using the open reading frames from 150 species. Scores at each codon were assessed, where 100% conservation corresponds to a score of 1, and this score also receives the addition of 0 if dN-dS of the site is below the mean, addition of 0.25 for sites with values above the mean to 1 standard deviation above the mean, addition of 0.5 for sites greater than 1 standard deviation but below 2 standard deviations, and addition of 1 for sites greater than 2 standard deviations. Therefore, a score of 2 is maximal suggesting an amino acid that is 100% conserved with codon wobble indicative of a high selection rate at the position. (B) Conservation values were placed on a 21-codon sliding window (combining values for 10 codons before and after each position) to identify conserved motifs within the gene. (C) Model of PEA15 protein with a structural z-score of 0.12 (assessed with YASARA2 knowledge-based force field) suggesting accurate predictions of fold space. Colors are based on 150 species alignments fed into ConSurf. (PDF) [file pgen.1008671.s010.pdf]

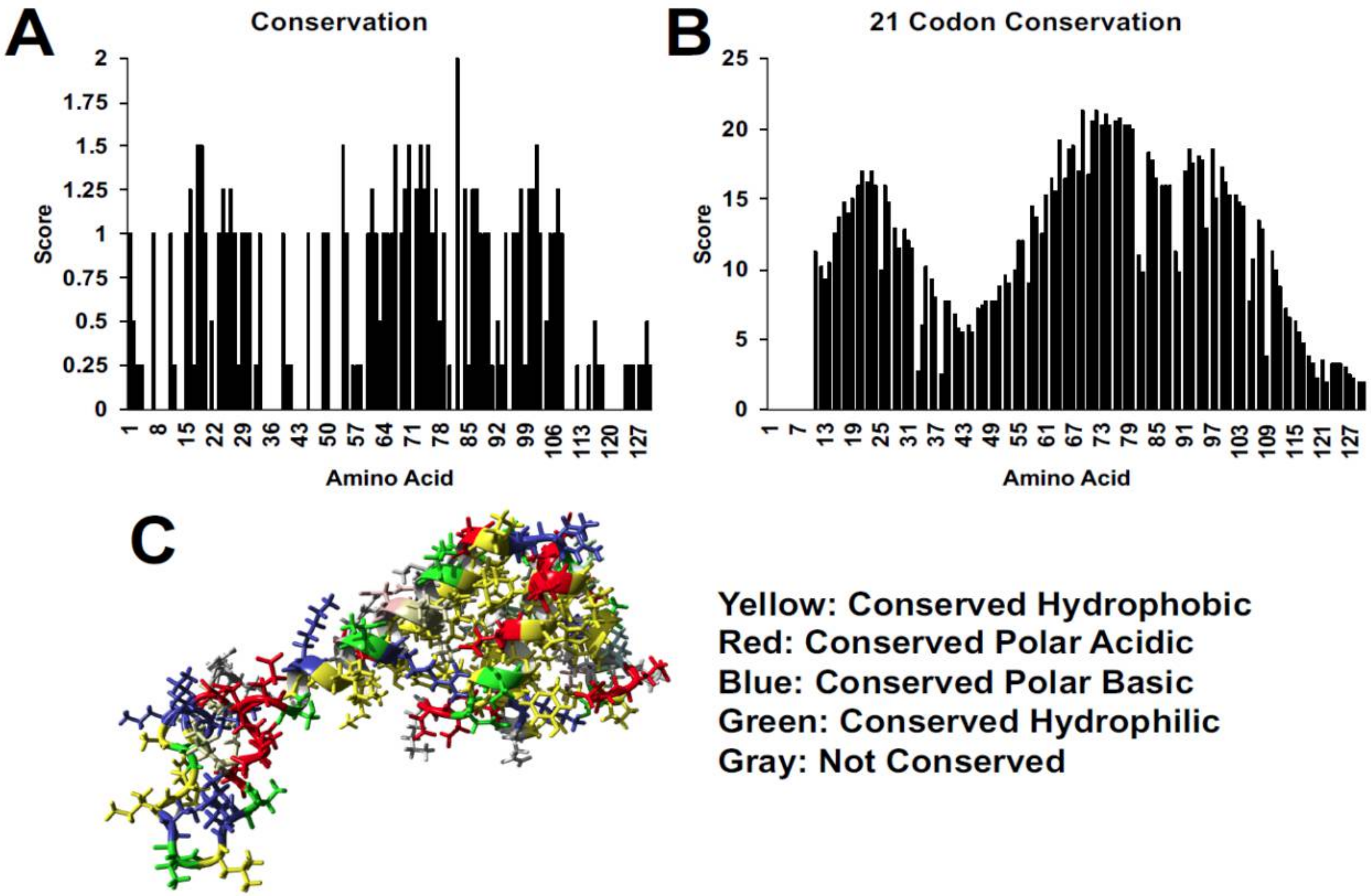

S4 Fig. PEA15 Conservation. (**A**) Conservation of PEA15 gene sequence was performed using the open reading frames from 150 species. Scores at each codon were assessed, where 100% conservation corresponds to a score of 1, and this score also receives the addition of 0 if dN-dS of the site is below the mean, addition of 0.25 for sites with values above the mean to 1 standard deviation above the mean, addition of 0.5 for sites greater than 1 standard deviation but below 2 standard deviations, and addition of 1 for sites greater than 2 standard deviations. Therefore, a score of 2 is maximal suggesting an amino acid that is 100% conserved with codon wobble indicative of a high selection rate at the position. (**B**) Conservation values were placed on a 21-codon sliding window (combining values for 10 codons before and after each position) to identify conserved motifs within the gene. (**C**) Model of PEA15 protein with a structural z-score of 0.12 (assessed with YASARA2 knowledge-based force field) suggesting accurate predictions of fold space. Colors are based on 150 species alignments fed into ConSurf.
