# Supplementary material for: PEA15 loss of function and defective cerebral development in the domestic cat
Source: PLoS Genet. 2020 Dec 8;16(12):e1008671. doi: 10.1371/journal.pgen.1008671 (PMC7723247; doi:10.1371/journal.pgen.1008671)
Supplement: S5 Fig — Out of 16 original cats, 1 cat was excluded from further analysis because of death by grand mal seizure, evident by PCA. 3 cats were excluded from further analysis because they were kittens to avoid detection of developmental false positive signals in differential expression analysis (1 kitten was also an outlier by PCA). 2 cats were excluded on the basis of being clear outliers by PCA. 10 cats (4 homozygous mutant, 3 heterozygous mutant, and 3 homozygous non-mutant) remained for analysis. (PDF) [file pgen.1008671.s011.pdf]

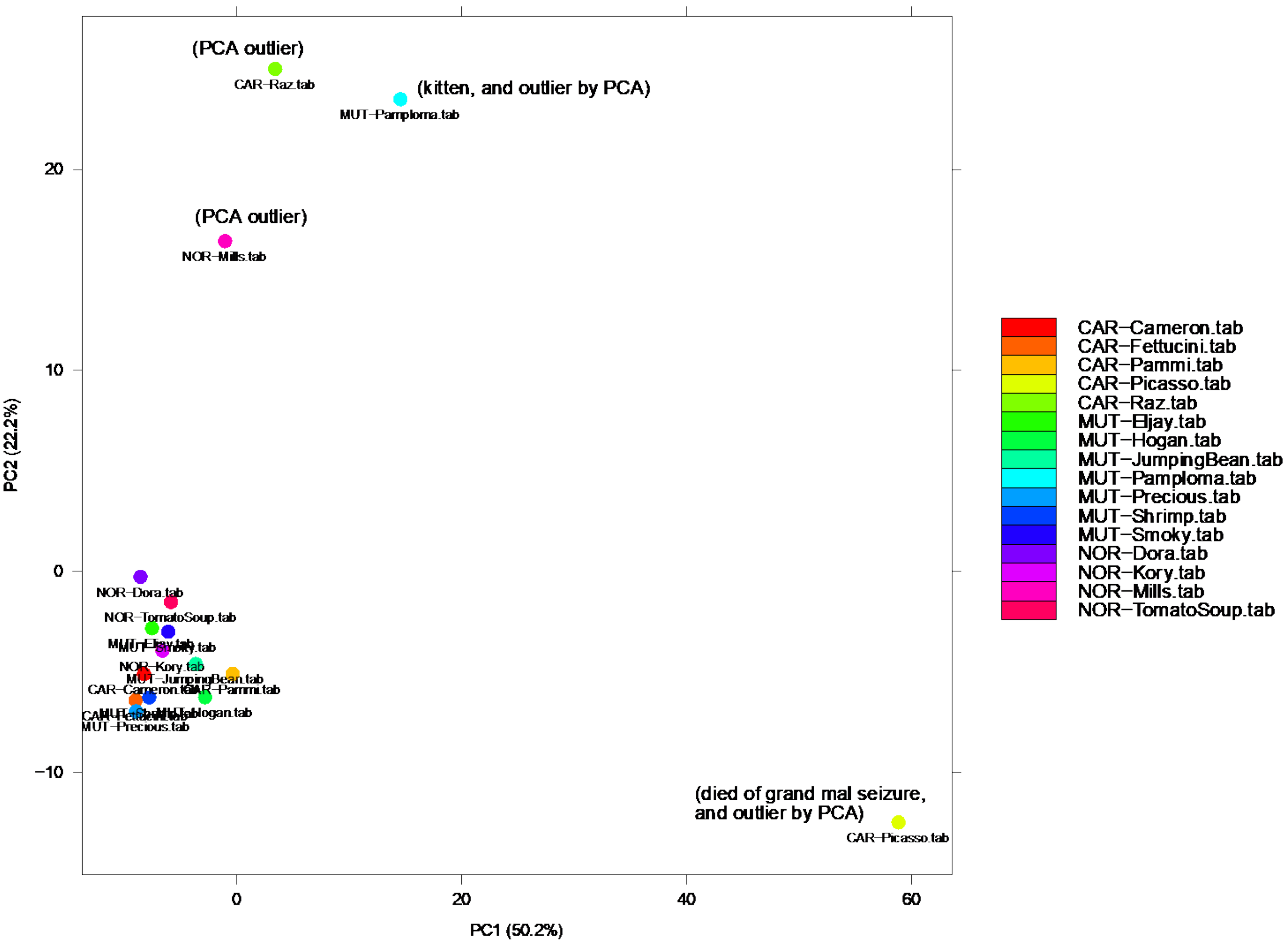

S5 Fig. Principal component analysis (PCA) of RNA-seq data from cat cortex. Out of 16 original cats, 1 cat was excluded from further analysis because of death by grand mal seizure, evident by PCA. 3 cats were excluded from further analysis because they were kittens to avoid detection of developmental false positive signals in differential expression analysis (1 kitten was also an outlier by PCA). 2 cats were excluded on the basis of being clear outliers by PCA. 10 cats (4 homozygous mutant, 3 heterozygous mutant, and 3 homozygous non-mutant) remained for analysis.
