# Supplementary material for: PEA15 loss of function and defective cerebral development in the domestic cat
Source: PLoS Genet. 2020 Dec 8;16(12):e1008671. doi: 10.1371/journal.pgen.1008671 (PMC7723247; doi:10.1371/journal.pgen.1008671)
Supplement: Attachment — Submitted filename: PEA15_Reply_To_Reviewers.docx [file pgen.1008671.s012.docx]

**We thank the reviewers for the thorough review of the manuscript and the helpful comments and suggestions. Below we provide (in bold) a point-by-point response to each reviewer’s comments.**

Reviewer #1: The authors report the discovery of a recessive mutation in cats that leads to severe alterations in brain structure and function that produces a variety of associated traits for which abundant phenotypic data is provided at behavioral, anatomical and microanatomic levels. Interestingly, the affected feline gene is PEA15 in which knockout mice models show no structural alterations. In vitro studies of fibroblasts support increased apoptosis and proliferation of other cell types and implicate PEA15 function in these pathways.

The identification of the likely causal variant from WGS and RNAseq as presented within Fig 3 and the text is complete and well done, as is the sequence, expression and protein quantitation alteration presented in Fig 4.

The abundance of supporting data for the phenotypic effects is far beyond many/most domestic animal model genetic investigations. I am not an expert in any aspect of the neuroanatomy or function, but am very much inclined to trust the author’s conclusions. Further the in vitro data indicating PEA15 is involved in intracellular signaling pathways is quite sufficient as well. The discussion is also well-presented and succinct.

My only comment is in regards to the 25 cats from the colony had the cerebral dysgenesis phenotype in which genotyping for 2 lysosomal storage disease mutations showed that the CB locus is not co-segregating. In and around line 102 please clearly state how many of the 25 CD affected cats were homozygous for the GM2A and the MPSVI wild type alleles. And how many were homozygous for both wild type alleles.

**We added text to state explicitly how many cerebral dysgenesis cats were homozygous wild type for GM2A (21 of 25), MPSVI (16 of 25), and both (12 of 25).**

Reviewer #2: This submission impressively illustrates the current possibilities of WGS-based precision medicine in cats. The success rate of WGS-based identification of rare disease-causing genes is difficult to determine because about two-thirds of studied human diseases discover too many credible candidate variants and in the remaining one-third there are none. It is obviously of advantage to study rare conditions in purebred companion animals like cat. This in combination with the aspect of potential model usage for human research instead of the standard rodent model highlights the value of this work. The observed brain anomaly is specific to mammals with gyrifcation that is not present in mice or rats.

As a non-expert in both feline medicine and neuroanatomy I would like to restrict my comments on the positional cloning which is somehow representing the core of this submission. The genome-wide mapping is difficult to read, and although the fine-mapping on F1 (Figure 3B) looks very convincing it could be presented in the supplement. Also the data illustrating the results of the additionally performed linkage analysis could be moved to the supplement (Figure 3C+D) as it looks somehow redundant. The confirmation of the two mapping approaches are written in the text, I guess that is sufficient in the light of the entire length of the manuscript with many nice figures. WGS data filtering was based on the assumption of autosomal recessive inheritance but focussed only on protein-changing variants. Please specify “all variants” mentioned in line 151 with detailed numbers, e.g. in supplementary table. Furthermore the chosen mapping approach based on homozygosity is not clearly presented as the resolution of Figure 3A is poor. A table reporting all shared ROHs across the entire genome would be more informative. Furthermore the possibility of biallelic pathogenic variants was not considered (compound heterozygosity). During the fine-mapping based on amplicon-resequencing it looks like that only protein-changing variants were considered (lines 155-158 and 164-169), some more details would be nice to have. Why all non-coding variants were ruled out at this stage? In addition, it is not quite clear why only the 1.3Mb region on F1 was further studied. Why it was allowed to assume that the disease-causing variant is fully absent from the 99 Lives variant catalogue?

**We apologize that the genome-wide mapping in Figure 3A is difficult to read. To address this, we are providing 1200 dpi tiff files, and also are including more clarification in both the main text and the figure legend on how to interpret Figure 3A.**

**With regard to the suggestion that Figure 3B-D could be moved to the supplement, we note that the fine-mapping data in Figure 3B is based on haplotype and association analysis. Together with the pedigree-based formal linkage analysis in 3C, and the variant annotation in 3D (below), these complementary (but not redundant) approaches provide rigorous support for the central claim regarding the genotype-phenotype relationship. Because rigor of genetic inference is a critical aspect of PLOS Genetics, we prefer to keep Figure 3 in the main section of the manuscript, but to explain its importance more clearly as described further below, and in the revised text.**

**With regard to the question about genotyping coding variants, we did indeed choose coding variants to genotype for fine-mapping, the purpose of which was not as a WGS filter but as a tool to narrow the initial WGS-based association region of 5 Mb (Figure 3A) to a 1.3 Mb critical interval (Figure 3B). All variation within this 1.3 Mb interval, including non-coding variation and copy number variation, was considered as potentially causal, and we have explained this point more clearly in the revised text. This is part of the importance of Figure 3D, which presents damage prediction scores for all variants in this region, including non-coding variation (white circles).**

**In the revised manuscript, we list all variants that meet filtering criteria (homozygous in affected, heterozygous in obligate carriers), coding and non-coding, in a new supplementary table (Table S4).**

**With regard to the questions about compound heterozygosity and filtering criteria that excluded all 99Lives variants, we began with the hypothesis that the cause of cerebral dysgenesis was a private variant unique to the Auburn research colony, that there was only a single variant, and therefore affected animals would all be homozygous. That hypothesis is based on the observation that the cerebral dysgenesis phenotype was observed in a closed breeding colony that has been isolated for nearly 50 years, and there are no other reports of similar phenotypes in the domestic cat pathology literature. The linkage and association data proves that affected animals are homozygous for the same haplotype. It is formally possible that additional variants on that haplotype (that might exist in the 99Lives collection) could be causal (when homozygous or as compound heterozygotes); however, even with this relaxed filtering criteria, only the PEA15 variant exhibits an elevated CADD score. Our strategy and analysis pipeline is explained more clearly in the revised manuscript, in which we present all variants in the critical region (coding and non-coding) with an allele frequency < 5% in the 99Lives collection.**

Minor comments:
Please strictly adhere to the published guidelines of sequence variant nomenclature (<http://varnomen.hgvs.org/>) e.g. lines 85-86 of line 165.

**Done.**

Avoid talking about mutations, maybe variant would be more appropriated. Finally, please also adhere to the guidelines for the interpretation of sequence variants (<https://www.nature.com/articles/gim201530>). Accordingly the LoF variant could be classified as “pathogenic”.

**Done.**

The variant details should also be presented in Figure 4A.

**Done.**

Within the first section of the results it would good to refer to the OMIA database (<https://omia.org/>) to refer to the tested variants in GM2A and MPSVI.

**We checked OMIA, and for particular entries, OMIA recommends instead to cite the original journal article, which we have done.**

Reviewer #3: PEA15 LOSS OF FUNCTION AND DEFECTIVE CEREBRAL DEVELOPMENT
IN THE DOMESTIC CAT

Graff and coworkers have identified a loss-of function mutation in the feline PEA15 gene that appears to cause defective cerebral development in the domestic cat. The affected cats exhibited impaired cerebral cortical expansion and folding as well as microcephaly. In particular, the cerebral cortex showed abnormalities. To investigate to genetic causes underlying the cerebral dysgenesis, the authors performed whole genome sequencing of eight affected cats and six obligate carriers and used conditional filtering for identified genetic variants under the assumption of an autosomal recessive mode of inheritance. RNA-seq was also done from cerebral cortex from four affected cats, three obligate carriers and three “non-mutant” cats.

GENERAL COMMENTS

This is an impressive study with a thorough analysis of a large amount of data and the resulsts will significantly contribute to the biological understanding of the observed abnormalities in the development of the cerebral cortex. A significant portion of the manuscript is focused on the pathology of the condition. This is very important when defining a condition. However, for most readers of PLOS Genetics, the pathological and histological results may be difficult to follow. A more explanatory description would help the reader with the interpretation of some of the histology. For example, Figure 6 includes six layers plus the white matter (WM). For an untrained eye and for non-experts in neuro-pathology, it will be difficult to observe the six layers and to know what constitutes the layers. I would therefore urge the authors to consider being more descriptive and explain the histology and the pathology for non-pathologist.

**For clarity, we have added the following explanation for the neuropathology in the revised manuscript: “In mammalian species, the cerebral cortex has a complex histological arrangement with six unique cortical layers that comprise different cell types: Layer I, known as the molecular, is closest to the cortical meningeal surface and contains predominately neuropil; Layers II and IV contain small stellate neurons; Layers III and V contain large pyramidal neurons and Layer VI contains fusiform neurons. These layers are most easily visualized in primates, and range from variably distinct in species such as the domestic cat to indistinct in rodents. Within the six layers, there are projection neurons, which mostly arise from Layers III and V and extend to form white matter (WM) tracts.”**

The genetic analyses including whole-genome sequencing, the RNA-seq as well as the bioinformatics is very well described and the conclusions are well supported. It is particularly fortunate that the authors have access to a sufficient number of individuals and tissues to perform a robust experimental set up and analyses.

In short, the conclusions of this study, including that the identified deletion in PEA15 is likely responsible for cerebral dysgenesis in the investigated domestic cats, is well supported by the Graff and co-workers.

MINOR REVISIONS AND COMMENTS

Please check the text under Table 1. “Of the two variants that change coding sequence, the missense variant in LY9 is not predicted to be damaging, and LY9 is not expressed in brain.”

**We have rewritten the sentence in the revised manuscript to make it more understandable.**

In the results section under “RNA-seq analysis” the sentence below with “unremarkable phenotypically” sounds somewhat strange. It is clear what you mean but please check the language. “Given minimal differences by RNA-seq and that heterozygous mutant cats are unremarkable phenotypically, we also performed a comparison of all 6 unaffected cats vs. homozygous mutant cats, revealing 25 differentially expressed (FDR<0.05) genes.”

**We have rewritten this phrase in the revised manuscript to make it more understandable.**

Table S3. Why is it question marks after the heading “On Figure?” and “PEA15 Variant?”? Please clarify.

**The question marks were meant to indicate that if the column read “Yes” that the variant(s) were either on the main figure or is the PEA15 variant. However, this is clear without the presence of the question marks as well, so we have removed them in the revised manuscript.**
